# Differential impact of individual autonomic domains on clinical outcomes in Parkinson’s disease

**DOI:** 10.1007/s00415-022-11221-9

**Published:** 2022-06-16

**Authors:** Katherine Longardner, Aristide Merola, Irene Litvan, Alberto Maria De Stefano, Simona Maule, Fabrizio Vallelonga, Leonardo Lopiano, Alberto Romagnolo

**Affiliations:** 1 Department of Neurosciences, University of California San Diego, 9500 Gilman Dr. MC 0886, La Jolla, CA 92093 USA; 2 Department of Neurology, Wexner Medical Center, Ohio State University, 395 W. 12th Ave., Columbus, OH 43210 USA; 3 Department of Neuroscience “Rita Levi Montalcini”, University of Turin, Via Cherasco 15, 10126 Turin, Italy; 4 Neurology 2 Unit, A.O.U. Città Della Salute e Della Scienza di Torino, Via Cherasco 15, 10126 Turin, Italy; 5 Department of Medical Sciences, Internal Medicine Division, Autonomic Unit and Hypertension Unit, University of Turin, Turin, Italy

**Keywords:** Parkinson’s disease, Dysautonomia, Orthostatic hypotension, Dementia, Disease milestones

## Abstract

**Introduction:**

While autonomic failure is a well-known prognostic factor for more aggressive disease progression in Parkinson’s disease (PD), with a three- to sevenfold higher risk of dementia and death within 10 years after the diagnosis, the individual impact of cardiovascular, gastrointestinal, urogenital, thermoregulatory, and pupillomotor autonomic domains on PD clinical outcomes remains unclear.

**Objectives:**

We sought to determine the 5-year risk of developing dementia, falls, postural instability, dysarthria, and dysphagia in PD patients with and without autonomic impairment at baseline and to assess the joint and individual association of each autonomic domain on these key functional outcomes. In addition, we aimed to determine the impact of each autonomic domain on activities of daily living (ADLs) and health-related quality of life (HRQoL).

**Methods:**

We enrolled 65 consecutive PD patients in a 5-year cohort study involving standardized evaluations of autonomic symptoms, orthostatic hypotension, and motor and non-motor features, including cognitive function. Associations were estimated as odds ratio and adjusted for PD duration, age, and baseline motor impairment.

**Results:**

Cardiovascular dysautonomia was associated with a sevenfold higher risk of developing dementia (95%CI: 1.154–50.436; *p* = 0.035) and a fivefold higher risk of falls (95%CI: 1.099–18.949; *p* = 0.039), as well as significantly higher impairment in ADLs (*p* = 0.042) and HRQoL (*p* = 0.031). No relevant associations were found between the other autonomic domains and these outcomes.

**Conclusions:**

Cardiovascular dysautonomia, but not other domains, showed an association with worse 5-year clinical outcomes in PD. Our data suggest a specific role for cardiovascular autonomic dysregulation in the pathogenic mechanisms of PD progression.

**Supplementary Information:**

The online version contains supplementary material available at 10.1007/s00415-022-11221-9.

## Introduction

It has been estimated that 50–70% of individuals with Parkinson’s disease (PD) experience disturbances from autonomic nervous system failure, including cardiovascular, urogenital, gastrointestinal, thermoregulatory, and pupillomotor dysfunction [1–3]. Previous studies showed that general dysautonomia, and neurogenic orthostatic hypotension (OH) in particular, are associated with negative health outcomes in PD, including faster motor and cognitive decline, more frequent falls and hospitalizations, and a higher risk of developing dementia, disability, and death [4–7]. Still, the individual impact of each autonomic domain on disease progression and whether autonomic disturbances contribute directly to worse outcomes (e.g., extreme hemodynamic fluctuations potentially causing repeated cerebral hypoperfusion) or are merely associated with them (i.e., a marker of a more aggressive disease phenotype) remains unclear [8, 9].

In this 5-year prospective observational study, our primary aim was to determine the individual and joint association of each autonomic domain on developing critical PD disability milestones, including dementia, falls, postural instability, dysarthria, and dysphagia. Our secondary aim was to determine the impact of autonomic domains on progression of activities of daily living (ADLs) and health-related quality of life (HRQoL) impairment. Furthermore, we investigated the association between clinically defined OH and these outcomes.

## Methods

### Study design

This single-center, prospective, observational cohort study aimed to evaluate the impact of autonomic symptoms on disability milestones and functional outcomes in PD. Consecutive participants were enrolled from the Movement Disorders Center of the University of Torino between April 2015 and March 2016. The local institutional review board approved the study, and all participants gave written informed consent.

### Eligibility criteria

Inclusion criteria were as follows: PD diagnosed according to the United Kingdom Brain Bank Criteria [10]; disease duration at least two years at baseline; age between 18 and 80 years; and stable dose of dopaminergic therapy and/or antihypotensive or antihypertensive medications for at least 4 weeks. Exclusion criteria were neurological signs suggestive of a diagnosis other than idiopathic PD; diabetes mellitus or other condition associated with autonomic neuropathy [11]; cardiac arrhythmia or coronary artery disease, cardiac valve disease or clinically relevant cardiac structural abnormalities; severe chronic renal insufficiency (glomerular filtration rate < 30 ml/min); undergoing chemotherapy; monoclonal gammopathy of uncertain significance (MGUS); major psychiatric disorder in accordance with Diagnostic and Statistical Manual of Mental Disorders (DSM) 5th ed. Criteria (American Psychiatric Association, 2013); or treatment with alpha-adrenergic antagonists for prostatic disorders.

### Baseline assessments

Clinical and demographic data at baseline included age, medical history, and duration of PD from symptom onset. All medications were recorded and the levodopa equivalent daily dose (LEDD) calculated according to the conversion table proposed by Tomlisonet al. [12].

Clinical rating scales included motor assessment using the Movement Disorder Society-Unified Parkinson's Disease Rating Scale (MDS-UPDRS) Part III (motor examination) [12] during the best “on” state, defined as the period of perceived maximal efficacy from dopaminergic medications. Non-motor symptoms were assessed using the Non-Motor Symptoms Scale (NMSS) [13].

Autonomic and blood pressure (BP) assessments included the validated 25-item Scale for Outcomes in Parkinson’s Disease-Autonomic (SCOPA-AUT), a one-dimensional linear scale with high internal construct validity [14]. The following SCOPA-AUT subscales were independently rated: gastrointestinal (items 1–7); urogenital (8–13, for men 22–23 and for women 24–25); cardiovascular (14–16); thermoregulatory (17–18, 20–21); and pupillomotor (19). The impairment of an autonomic domain was defined when according to symptoms experienced in the past month at least one of the related items was rated ≥ 2; wherein 0, never; 1, sometimes; 2, regularly; and 3, often [15].

Patients underwent BP measurements using an automated sphygmomanometer (HEM-7200—Omron Healthcare Co. Kyoto, Japan) placed at heart level on the left arm, in the following conditions: (a) while sitting in a chair after at least 10 min of rest; (b) after a minimum of 10 min of supine rest; and (c) after one and three minutes of active standing. To minimize BP variability due to antiparkinsonian medications, patients were evaluated in standardized conditions. BP assessments were performed in the morning in the best “on” state, and at least 3 h after a meal. OH was defined as a BP drop ≥ 20 mmHg systolic or 10 mmHg diastolic within 3 min of standing from a supine position [16]. Patients with a rise in heart rate (HR)/fall in systolic BP ratio > 0.5 beats per minute (bpm)/mmHg [17] were considered as having non-neurogenic OH (i.e., due to iatrogenic cause, dehydration, venous pooling in the lower limbs, cardiac failure, etc.) and were excluded from the OH analyses. Hemodynamically relevant OH was defined as orthostatic mean arterial pressure (MAP) ≤ 75 mmHg [18]. Supine hypertension (SH) was defined as supine systolic BP ≥ 140 mmHg or diastolic BP ≥ 90 mmHg in patients affected by neurogenic OH (nOH) [19].

Functional and cognitive assessments included the MDS-UPDRS Parts I (non-motor experience of daily living), II (motor experience of daily living), and IV (motor complications) [13]; Montreal Cognitive Assessment (MoCA; range 0–30, lower is worse) [20]; and the 8-item PD Quality of Life Questionnaire (PDQ-8 single index; range 0–100, higher is worse) [21].

### Follow-up assessments

After 5 years, the patients underwent the following assessments to evaluate functional outcomes, independence in ADLs, and HRQoL: MDS-UPDRS Parts I and II, MoCA, and PDQ-8; the number of falls in the previous 4 weeks.

Five specific disability milestones were chosen to define disease progression, including the following: (1) dementia, defined as MoCA score < 21/30 [22]; (2) falls in the 4 weeks prior to the evaluation; (3) postural instability, defined as a score ≥ 3 of item 3.12 of the MDS-UPDRS; (4) dysphagia, defined as a score ≥ 3 of item 2.3 of the MDS-UPDRS; and (5) dysarthria, defined as a score ≥ 3 of item 2.1 of the MDS-UPDRS.

### Outcome measures and statistical analyses

Our primary aim was to evaluate the 5-year risk of developing the five key disability milestones listed above in patients with and without PD-associated autonomic symptoms and with and without nOH at baseline, evaluating the individual and joint impact of each autonomic domain. Our secondary aim was to determine the role of autonomic dysfunction and nOH in progression of ADL and HRQoL impairment.

Primary endpoints were dementia, falls, postural instability, dysphagia, and dysarthria. A binary logistic regression was used to estimate the odds ratio (OR) of global autonomic impairment at baseline, as measured by the total SCOPA-AUT score, and of each individual autonomic domain impairment (independent variables) on the occurrence of disability milestones along the 5-year follow-up (dependent variables), adjusting for age, disease duration, and motor symptom severity (MDS-UPDRS Part III) at baseline. The logistic regression was run twice as follows: (a) considering SCOPA-AUT and each autonomic domain separately (univariate); and (b) considering all the autonomic domains together (multivariate). Only for dementia, the analyses were run including the entire sample and, then again, excluding the individuals who had dementia at baseline. The same analyses were used to evaluate the association between nOH at baseline and disability milestones, and between hemodynamically relevant nOH and disability milestones. A sub-analysis on the impact of SH associated with nOH was also included. The Hosmer and Lemeshow’s goodness-of-fit test was applied.

Secondary endpoints included impairment of ADLs, as measured by the MDS-UPDRS Parts I and II, and HRQoL, as measured by the PDQ-8. A repeated-measures ANOVA was used to evaluate progression independent variables (MDS-UPDRS Part I, Part II, Parts I + II combined, and PDQ-8), adjusting for age, disease duration, and motor symptom severity at baseline (covariates), between patients with and without the following conditions: (a) impairment in each autonomic domain; (b) nOH; and (c) hemodynamically relevant nOH.

Clinical and demographic characteristics were summarized as mean ± standard deviation and range or absolute number and percentages, as appropriate. The differences between SCOPA-AUT, MDS-UPDRS Parts I and II, MoCA, and PDQ-8 scores at baseline and at 5-year follow-up were evaluated by means of the Wilcoxon non-parametric test. All the analyses were performed with Statistical Package for the Social Sciences (SPSS 27.0 for Macintosh, Chicago, IL), using two-tailed p-values with a level of significance of 0.05.

### Data availability

The data that support the findings of this study are available from the corresponding author, upon reasonable request.

## Results

Out of the 65 PD patients enrolled in the study, nine (15.3%) died during the observational study period (mean time from baseline: 29.7 ± 7.7 months), and six (9.2%) were lost to follow-up (Supplementary Table 1). Thus, 5-year follow-up data were available for 50 patients (Tables 1 and 2), and showed a 66.9% overall progression in global ADL impairment (MDS-UPDRS Parts I + II: from 23.1 ± 10.3 to 38.6 ± 17.8; p < 0.001) and a significant 81.4% worsening in HRQoL (PDQ-8: from 22.6 ± 19.0 to 41.0 ± 29.4; *p* < 0.001). MoCA score significantly declined by 12.3% (from 26.1 ± 3.5 to 22.9 ± 5.4; *p* < 0.001), with the prevalence of demented patients rising from 8.0% (4/50) to 30% (15/50); the 5-year incidence of dementia was 23.9% (11/46).

**Table 1:** Demographic and clinical characteristics at baseline

Sex (males/females)	30/20 (60%/40%)
Age (years)	64.3 ± 9.4 (38–80)
Disease duration (years)	12.8 ± 6.1 (2–26)
MDS-UPDRS-I	12.0 ± 5.5 (3–30)
MDS-UPDRS-II	11.1 ± 5.8 (2–26)
MDS-UPDRS-III	28.2 ± 14.1 (3–66)
MDS-UPDRS-IV	3.8 ± 3.7 (0–14)
Hoehn and Yahr stage	2.5 ± 0.6 (2–4)
Total LEDD (mg)	935.9 ± 538.8 (100–2100)
NMSS	34.6 ± 24.2 (3–110)
SCOPA-AUT	13.1 ± 8.7 (0–34)
Gastrointestinal domain impairment (yes/no)	32/18 (64%/36%)
Urogenital domain impairment (yes/no)	34/16 (68%/32%)
Cardiovascular domain impairment (yes/no)	10/40 (20%/80%)
Thermoregulatory domain impairment (yes/no)	19/31 (38%/62%)
Pupillomotor domain impairment (yes/no)	11/39 (22%/78%)
Neurogenic OH (yes/no)	13/37 (26%/74%)
Hemodynamically relevant OH (yes/no)	10/40 (20%/80%)
Supine hypertension (yes/no)	6/7 (46.2%/53.8%)
MoCA	26.1 ± 3.5 (14–30)
Dementia (yes/no)	4/46 (8%/92%)
PDQ-8 single index	22.6 ± 19.0 (0.0–81.2)

Results are reported as mean ± standard deviation (range) or absolute values (percentage), as appropriate. Supine hypertension refers only to patients affected by neurogenic OH

LEDD, Levodopa Equivalent Daily Dose; MDS-UPDRS, Movement Disorders Society Unified Parkinson’s Disease Rating Scale; MoCA, Montreal Cognitive Assessment; NMSS, Non-Motor Symptom Scale; OH, Orthostatic Hypotension; PDQ-8, Parkinson’s Disease Questionaire-8; SCOPA-AUT, Scale for Outcomes in Parkinson’s Disease-Autonomic

**Table 2:** Demographic and clinical characteristics at 5-year follow-up

Sex (males/females)	30/20 (60.0%/40.0%)
Age (years)	69.3 ± 9.4 (43–85)
Disease duration (years)	17.8 ± 6.1 (6–31)
MDS-UPDRS-I	16.2 ± 8.2 (1–36)
MDS-UPDRS-II	22.4 ± 11.4 (3–44)
MDS-UPDRS-IV	6.5 ± 5.9 (0–24)
Total LEDD (mg)	960.0 ± 484.9 (200–2150)
NMSS	74.4 ± 55.9 (9–246)
SCOPA-AUT	21.3 ± 10.4 (6–48)
MoCA	22.9 ± 5.4 (10–29)
Dementia (yes/no)	15/35 (30.0%/70.0%)
Falls (yes/no)	31/19 (62.0%/38.0%)
Dysphagia (yes/no)	13/37 (26.0%/74.0%)
Postural instability (yes/no)	28/22 (56.0%/44.0%)
Dysarthria (yes/no)	18/32 (36.0%/64.0%)
PDQ-8 single index	41.0 ± 29.4 (0.0–93.7)

Results are reported as mean ± standard deviation (range) or absolute values (percentage), as appropriate

LEDD, Levodopa Equivalent Daily Dose; MDS-UPDRS, Movement Disorders Society Unified Parkinson’s Disease Rating Scale; MoCA, Montreal Cognitive Assessment; NMSS, Non-Motor Symptom Scale; PDQ-8, Parkinson’s Disease Questionaire-8; SCOPA-AUT, Scale for Outcomes in Parkinson’s Disease-Autonomic

The vast majority of patients (86.0%) reported a significant progression in their baseline autonomic symptoms over follow-up, with the SCOPA-AUT total score increasing from 13.1 ± 8.7 at baseline to 21.3 ± 10.4 points at year 5 (62.6% worsening) (*p* < 0.001). The gastrointestinal domain worsened by 70.3% (from 3.7 ± 2.9 to 6.3 ± 3.8; *p* < 0.001), the urogenital by 55.4% (from 5.6 ± 5.2 to 8.7 ± 5.2; *p* < 0.001), the cardiovascular by 66.7% (from 1.2 ± 1.9 to 2.0 ± 2.3; *p* < 0.001); the thermoregulatory by 54.5% (from 2.2 ± 2.5 to 3.4 ± 3.1; *p* = 0.008), and the pupillomotor by 125% (from 0.4 ± 0.7 to 0.9 ± 1.0; *p* = 0.002).

### Dysautonomia and disability milestones

The total SCOPA-AUT score was not associated with any of the disability milestones evaluated: dementia (*p* = 0.478), falls (*p* = 0.790), postural instability (*p* = 0.326), or dysphagia (*p* = 0.253), with a trend towards statistical significance only for dysarthria (*p* = 0.061) (Table 3). However, the cardiovascular score was independently associated with a higher risk of developing dementia, both in the univariate and in the multivariate analysis (OR: 9.058; 95% CI: 1.548–53.000; *p* = 0.014; and OR: 7.630; 95% CI: 1.154–50.436; *p* = 0.035, respectively) (Table 3) and with falls in the univariate analysis (OR: 5.701; 95% CI: 1.099–18.949; *p* = 0.039) (Table 3). None of the autonomic domains were associated with postural instability, dysphagia, or dysarthria (Table 3).

**Table 3:** Analysis of association between dysautonomia and disability milestones

Dysautonomic features	Univariate		Multivariate	
	OR (95%CI)	*p* value	OR (95%CI)	*p* value
Dementia				
SCOPA-AUT (total score)	1.032 (0.948–1.123)	0.470	–	–
Gastrointestinal domain impairment	3.123 (0.480–20.310)	0.233	2.186 (0.221–21.603)	0.503
Urogenital domain impairment	0.541 (0.105–2.777)	0.462	0.493 (0.066–3.660)	0.489
Cardiovascular domain impairment	8.473 (1.476–48.638)	**0.017**	6.955 (1.113–43.475)	**0.038**
Thermoregulatory domain impairment	3.843 (0.699–12.089)	0.131	2.055 (0.324–13.043)	0.445
Pupillomotor domain impairment	1.327 (0.231–7.634)	0.751	0.988 (0.115–8.488)	0.991
After removing patients with dementia at baseline				
SCOPA-AUT (total score)	1.032 (0.946–1.126)	0.478	–	–
Gastrointestinal domain impairment	2.409 (0.382–15.211)	0.350	1.424 (0.138–14.739)	0.767
Urogenital domain impairment	0.414 (0.077–2.220)	0.303	0.383 (0.049–2.973)	0.359
Cardiovascular domain impairment	9.058 (1.548–53.000)	**0.014**	7.630 (1.154–50.436)	**0.035**
Thermoregulatory domain impairment	4.389 (0.822–19.834)	0.091	2.281 (0.286–18.164)	0.436
Pupillomotor domain impairment	1.599 (0.279–9.179)	0.599	1.190 (0.137–10.370)	0.875
Falls				
SCOPA-AUT (total score)	1.011 (0.932–1.096)	0.790	–	–
Gastrointestinal domain impairment	2.028 (0.489–8.401)	0.330	1.417 (0.278–7.232)	0.675
Urogenital domain impairment	1.255 (0.310–5.084)	0.750	1.196 (0.250–5.712)	0.823
Cardiovascular domain impairment	5.701 (1.099–18.949)	**0.039**	4.294 (0.413–44.609)	0.222
Thermoregulatory domain impairment	1.672 (0.425–6.581)	0.462	1.639 (0.291–9.226)	0.575
Pupillomotor domain impairment	0.328 (0.268–1.588)	0.266	0.290 (0.051–1.656)	0.164
Dysphagia				
SCOPA-AUT (total score)	1.069 (0.953–1.199)	0.253	–	–
Gastrointestinal domain impairment	10.093 (0.641–58.872)	0.100	13.352 (0.793–65.771)	0.072
Urogenital domain impairment	1.025 (0.257–3.622)	0.539	0.810 (0.113–5.554)	0.755
Cardiovascular domain impairment	4.808 (0.601–38.447)	0.139	6.082 (0.546–44.745)	0.142
Thermoregulatory domain impairment	1.285 (0.246–6.700)	0.766	0.416 (0.051–3.417)	0.414
Pupillomotor domain impairment	0.626 (0.070–5.626)	0.676	0.757 (0.047–12.092)	0.844
Dysarthria				
SCOPA-AUT (total score)	1.080 (0.996–1.170)	0.061	–	–
Gastrointestinal domain impairment	4.375 (0.874–21.904)	0.073	3.160 (0.552–18.082)	0.196
Urogenital domain impairment	2.883 (0.640–9.990)	0.168	2.898 (0.539–15.588)	0.215
Cardiovascular domain impairment	2.980 (0.680–13.053)	0.147	2.226 (0.453–10.933)	0.325
Thermoregulatory domain impairment	1.599 (0.463–5.520)	0.457	1.310 (0.280–6.129)	0.732
Pupillomotor domain impairment	1.121 (0.250–5.026)	0.882	1.248 (0.224–6.943)	0.800
Postural instability				
SCOPA-AUT (total score)	1.040 (0.961–1.126)	0.326	–	–
Gastrointestinal domain impairment	1.853 (0.470–7.298)	0.378	1.204 (0.259–5.602)	0.813
Urogenital domain impairment	1.268 (0.331–4.851)	0.729	1.551 (0.345–6.968)	0.567
Cardiovascular domain impairment	2.928 (0.513–16.727)	0.227	2.181 (0.358–13.306)	0.398
Thermoregulatory domain impairment	2.462 (0.661–9.174)	0.179	2.331 (0.492–11.055)	0.286
Pupillomotor domain impairment	1.112 (0.252–4.908)	0.888	0.955 (0.191–4.782)	0.955

Results are reported as Odds Ratio (OR); 95% Confidence Interval is reported in brackets. Analyses were corrected for age, disease duration, and motor impairment (MDS-UPDRS-III) at baseline

Bold indicates the significant value of *p*-value: statistical significance

SCOPA-AUT, Scale for Outcomes in Parkinson’s Disease-Autonomic

### Neurogenic OH and disability milestones

At baseline, 13/50 patients (28.8%) had clinically defined nOH (Table 1), with 10 of those patients (76.9%) meeting the criteria for hemodynamically relevant OH. Four patients (30.8%) with nOH did not report significant OH-related symptoms on the SCOPA-AUT evaluation; despite that, two of them (50%; one demented and one non-demented) had hemodynamically relevant nOH. Only one patient reported cardiovascular dysautonomia symptoms without having OH. Six out of 13 patients with nOH (46.2%) met the criteria for SH (Table 1). Finally, two patients had non-neurogenic OH. The presence of nOH at baseline, after correcting for age, disease duration, and motor impairment, was associated with dementia at the 5-year follow-up (OR: 5.213; 95% CI: 1.194–24.391; *p* = 0.036), even after removing patients already affected by dementia at baseline (OR: 5.153; 95% CI: 1.289–26.943; *p* = 0.029). This association was even stronger when considering patients with concomitant SH (OR: 8.265; 95% CI: 2.026–32.103; *p* = 0.012). nOH was also associated with falls (OR: 7.129; 95% CI: 1.212–41.921; *p* = 0.030), but concomitant SH did not have an influence on falls (OR: 5.732; 95% CI: 0.492–49.271; *p* = 0.492). No association was found with postural instability (*p* = 0.655), dysphagia (*p* = 0.086), or dysarthria (*p* = 0.641). Similar results were found when analyzing patients with hemodynamically relevant nOH, confirming a significant, and even stronger, association with the development of dementia at the 5-year follow-up both before (OR: 7.713; 95% CI: 1.305–45.599; *p* = 0.024) and after (OR: 8.750; 95% CI: 1.502–50.987; *p* = 0.016) removing patients with dementia at baseline, and with falls (OR: 12.391; 95% CI: 1.215–58.367; *p* = 0.034). Again, no association was found with postural instability (*p* = 0.714), dysphagia (*p* = 0.129), or dysarthria (*p* = 0.155).

### Dysautonomia, ADL impairment, and quality of life

**Fig. 1:**
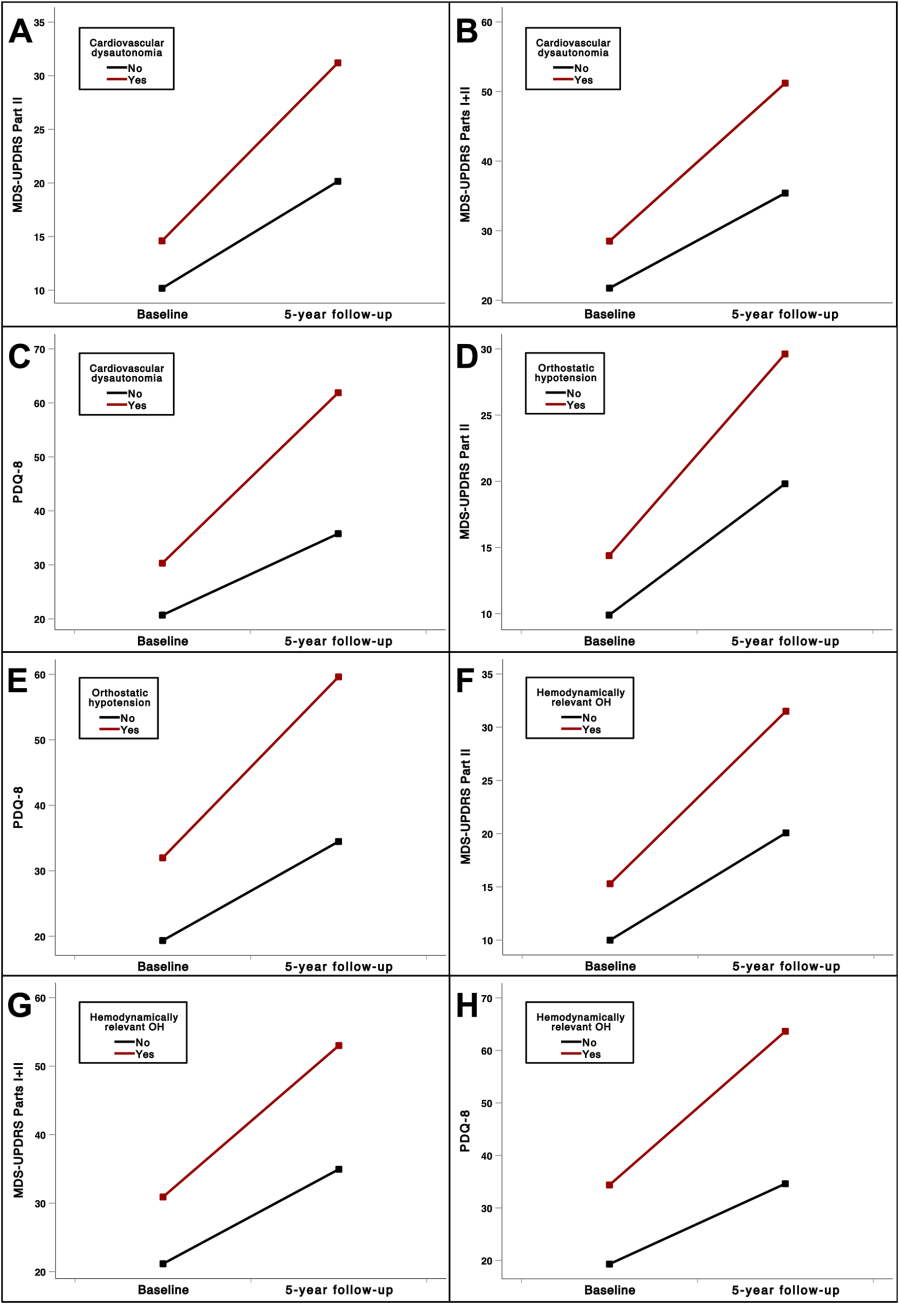
Five-year progression of activities of daily living and health-related quality of life impairment in patients with and without cardiovascular dysautonomia symptoms and patients with and without neurogenic orthostatic hypotension. Patients with cardiovascular dysautonomia symptoms showed greater worsening of motor experience of daily living (**A**), global experience of daily living (**B**), and health-related quality of life (**C**). Patients with orthostatic hypotension showed greater worsening of motor experience of daily living (**D**) and health-related quality of life (**E**). Patients with hemodynamically relevant orthostatic hypotension showed greater worsening of motor experience of daily living (**F**), global experience of daily living (**G**), and health-related quality of life (**H**). Covariates considered for ANCOVA analysis: age, disease duration, and MDS-UPDRS Part III at baseline. MDS-UPDRS, Movement Disorders Society Unified Parkinson’s Disease Rating Scale; PDQ-8, Parkinson’s Disease Questionaire-8

Among the different autonomic domains, only the cardiovascular domain was associated with a worse progression of functional disability. Patients with cardiovascular autonomic symptoms showed a greater worsening of the motor experience of daily living (MDS-UPDRS Part II, *F*: 4.187; *p* = 0.039) (Fig. 1A), the global experience of daily living (MDS-UPDRS Parts I + II, *F*: 3.786; *p* = 0.042) (Fig. 1B), and HRQoL (PDQ-8, *F*: 4.927; *p* = 0.031) (Fig. 1C).

### Neurogenic OH, ADL impairment, and quality of life

Patients with nOH showed worse function in the motor experience of daily living (*F*: 3.174; *p* = 0.048) (Fig. 1D) and HRQoL (*F*: 3.316; *p* = 0.045) (Fig. 1E).

When considering only hemodynamically relevant nOH, there remained a significant association with a worse progression of the motor experience of daily living (MDS-UPDRS Part II, *F*: 3.534; *p* = 0.043) (Fig. 1F), the global experience of daily living (MDS-UPDRS Parts I + II, *F*: 3.267; *p* = 0.046) (Fig. 1G), and HRQoL (*F*: 3.700; *p* = 0.042) (Fig. 1H).

## Discussion

In this 5-year prospective follow-up study comparing an outpatient cohort of 65 PD patients with and without autonomic impairment at baseline, we analyzed the contribution of general dysautonomia and individual autonomic domain dysfunction on disability milestones and progression of ADL impairment and HRQoL. Cardiovascular dysautonomia was associated with worse clinical outcomes at five years, including 7-times higher odds of dementia and 5-times higher odds of falls, and worse deterioration in functional impairment and HRQoL. These negative associations were confirmed when comparing patients with and without clinically defined nOH and further reinforced when considering the subset of patients with hemodynamically relevant nOH. No associations were found between global dysautonomia or any other autonomic domain and these outcomes.

Other studies have demonstrated a strong relationship between cardiovascular dysautonomia and disability in PD, including cognitive impairment, falls, postural instability, and functional decline [6, 9, 23]. A retrospective review of clinical data from 100 autopsy-confirmed PD patients revealed that earlier autonomic dysfunction, defined by more than one autonomic symptom documented for at least 6 months, was associated with 14% higher risk per year of developing any disability milestone, including falls, wheelchair dependence, cognitive impairment, dysphagia, dysarthria, and placement in a residential facility [7]. Moreover, a prospective study of 336 PD patients followed over 2 years found that baseline general dysautonomia (SCOPA-AUT total score) was associated with worse deterioration in HRQoL, assessed with the EuroQol Visual Analogue Scale [24]. However, neither of these studies investigated the association of individual autonomic domains.

In our study, we found that nOH was the major driver of disability progression and functional impairment in PD. Several theories exist to explain the pathophysiological mechanisms of this association. The “causative hypothesis” [25] posits that repeated episodes of cerebral hypoperfusion, often associated with increased BP variability and/or hypertensive events, damage the brain cumulatively, which causes cortical damage [26]. The causative hypothesis is supported by animal models of neurodegenerative dementia, where it has been suggested that hypoperfusion could impair regional brain microcirculation, reducing the delivery of energy substrates needed for proper neuronal function. This “critically attained threshold of cerebral hypoperfusion” could favor oxidative stress and mitochondrial abnormalities [27], and finally lead to the progression of metabolic and tissue pathology [28]. However, human studies on this topic are limited. In this context, we observed a higher incidence of dementia in patients with nOH and concomitant SH, suggesting a possible role of SH in promoting additional brain injury. However, this association should be interpreted with caution given our small sample size and contrasting results in the existing literature [26, 29]. Furthermore, managing individuals that have nOH and concomitant SH often presents a clinical dilemma given the narrow therapeutic window for blood pressure targets – since treating nOH can worsen SH and vice versa [30]. As confirmed in our study, the MAP seems to represent a key factor in predicting falls and OH complications: therefore, some extent of SH may be advisable when the MAP is excessively low (e.g., below 75 mmHg) [18].

An alternative theory, the “associative hypothesis”, suggests that dysautonomia, including cardiovascular dysfunction, is associated with a malignant phenotype of PD that progresses faster and manifests with rapid eye movement (REM) sleep behavior disorder and earlier development of cognitive impairment [31]. It is hypothesized that in this PD subtype, dysautonomia may occur due to diffuse alpha-synuclein pathology involving the central autonomic network, i.e., the insular cortex and brainstem as well as peripheral noradrenergic denervation [32]. However, these theories are not mutually exclusive. Extreme fluctuations in cerebral perfusion and neurodegeneration may have a synergistic effect on brain injury, leading to worse outcomes [33]. In a rat model of dementia, chronic hypoxic injury seems to accelerate the deposition of amyloid β in the frontal cortex and hippocampus and of hyperphosphorylated tau in the temporal cortex [34]. Furthermore, in PD patients with cardiovascular autonomic failure, arterial walls may become stiffer to enhance vasoconstriction in compensation for reduced noradrenaline release—increased arterial wall stiffness may contribute to cerebrovascular changes that associate with cognitive impairment and white matter lesions on magnetic resonance imaging [35].

Despite needing further validation, our findings seem to be more supportive of the “causative hypothesis”, since neither general autonomic impairment nor other individual autonomic domains were associated with worse outcomes in our cohort, as would be expected if dysautonomia was associated with the so-called “diffuse malignant” PD phenotype [31]. Furthermore, although we found an association between cardiovascular dysautonomia and falls, we did not find any association with postural instability. This discrepancy seems to suggest that cardiovascular dysautonomia is an independent risk factor for falls, even in the absence of a significant association with postural instability, which is another well-known risk factor for falls [36]. Whether this observation is due to the relatively small sample size or, on the contrary, suggests the predominant role of cardiovascular dysautonomia in the development of worse clinical outcomes, independently from the “associative hypothesis” of a widespread neurodegeneration, still needs further confirmation.

Although we did not find that any autonomic domains besides cardiovascular were associated with PD disability milestones, other longitudinal observational studies have shown that severity of general dysautonomia is associated with postural instability-gait disturbances motor phenotype in PD [37] and that gastrointestinal dysfunction is associated with worse cognition in early PD [38, 39]. However, these studies analyzed newly diagnosed PD patients, with a mean age at the disease onset greater than 60 years; therefore, their results are not fully comparable to ours and could reflect a phenotypic, rather than a causal, association.

Strengths of our study include the long-term prospective and longitudinal design, with detailed characterization of autonomic symptoms, and high retention rate. Limitations include the relatively small sample size and the monocentric design. Moreover, while the SCOPA-AUT is a robust and MDS-recommended rating scale for autonomic symptoms in PD, we acknowledge that its subjective nature has intrinsic limitations, which include the following: (a) difficulty for most patients in differentiating the "sometimes" and "regularly" responses [14], (b) the potential clinimetric weaknesses of dividing the scale into different autonomic domains [14, 40, 41]; (c) the limited capability in capturing pupillomotor and thermoregulatory dysfunction [42, 43]; and (d) the potential weak correlation between subjectively reported and objectively measured cardiovascular dysfunction [44–46]. Additionally, the use of the ΔHR/ΔBP ratio for the definition of nOH [17] may have excluded some patients with nOH. Finally, due to the COVID-19 pandemic, we were not able to repeat a complete motor examination and nOH assessment at the 5-year follow-up.

In summary, our results showed that among autonomic domains, cardiovascular dysautonomia was associated with a higher risk of developing disability milestones and worse progression of functional impairment and HRQoL over the course of 5 years. Whether cardiovascular dysautonomia directly causes worse outcomes in PD or is only one of the features of a multifaceted malignant phenotype remains a major knowledge gap. To clarify these hypotheses, and to determine the potential role of delaying, or even preventing, the development of dementia and other disability milestones by treating cardiovascular dysautonomia, additional prospective research in larger multicentric cohorts is needed.

## Supplementary Information

Below is the link to the electronic supplementary material.

Supplementary file1 (DOCX 17 kb)

## Data Availability

The data that support the findings of this study are available from the corresponding author, upon reasonable request.

## Declarations

### Data access and responsibility statement

A. Romagnolo has full access to all the data in the study and takes responsibility for the integrity of the data, the accuracy of the data analysis, and the conduct of the research.
