# Supplementary material for: Differential impact of individual autonomic domains on clinical outcomes in Parkinson’s disease
Source: J Neurol. 2022 Jun 16;269(10):5510–20. doi: 10.1007/s00415-022-11221-9 (PMC9201260; doi:10.1007/s00415-022-11221-9)
Supplement: Supplementary file 1 — Supplementary file1 (DOCX 17 kb) [file 415_2022_11221_MOESM1_ESM.docx]

**Supplementary Table 1: Demographic and clinical characteristics at baseline of the 15 patients excluded from the analyses (dead during follow-up, N= 9; lost to follow-up, N= 6)**

| **Patients excluded from the analyses** |  | **P value** |
| --- | --- | --- |
| **Sex (males/females)** | 10/5 *(66.7%/33.3%)* | .642 |
| **Age (years)** | 65.0 ± 5.5 *(55-72)* | .950 |
| **Disease duration (years)** | 13.1 ± 3.6 *(9-20)* | .901 |
| **MDS-UPDRS-I** | 11.4 ± 5.1 *(4-25)* | .607 |
| **MDS-UPDRS-II** | 13.5 ± 6.5 *(5-33)* | .132 |
| **MDS-UPDRS-III** | 29.7 ± 7.3 *(16-47)* | .518 |
| **MDS-UPDRS-IV** | 5.2 ± 4.4 *(0-13)* | .177 |
| **Hoehn and Yahr stage** | 2.7 ± 0.7 *(2-5)* | .257 |
| **Total LEDD (mg)** | 1111.6 ± 524.9 *(500-2000)* | .247 |
| **NMSS** | 32.9 ± 13.8 *(11-73)* | .586 |
| **SCOPA-AUT** | 13.7 ± 5.0 *(4-21)* | .548 |
| **Gastrointestinal domain impairment (yes/no)** | 9/6 *(60%/40%)* | .778 |
| **Urogenital domain impairment (yes/no)** | 9/6 *(60%/40%)* | .566 |
| **Cardiovascular domain impairment (yes/no)** | 4/11 *(26.7%/73.3%)* | .582 |
| **Thermoregulatory domain impairment (yes/no)** | 6/9 *(40%/60%)* | .889 |
| **Pupillomotor domain impairment (yes/no)** | 5/10 *(33.3%/66.7%)* | .371 |
| **Neurogenic OH (yes/no)** | 4/11 *(26.7%/73.3%)* | .959 |
| **Hemodynamically relevant OH (yes/no)** | 3/12 *(20%/80%)* | .374 |
| **Supine hypertension (yes/no)** | 2/4 *(50%/50%)* | .893 |
| **MoCA** | 25.8 ± 2.1 *(20-29)* | .227 |
| **Dementia (yes/no)** | 1/14 *(6.7%/93.3%)* | .865 |
| **PDQ-8 single index** | 25.4 ± 13.0 *(6.0-63.0)* | .140 |

Results are reported as mean ± standard deviation (*range*) or absolute values (percentage), as appropriate. Supine hypertension refers only to patients affected by neurogenic OH. P value: statistical differences (Chi square or Mann Whitney non-parametric test, as appropriate) vs. patients included in the study

LEDD: Levodopa Equivalent Daily Dose; MDS-UPDRS: Movement Disorders Society Unified Parkinson’s Disease Rating Scale; MoCA: Montreal Cognitive Assessment; NMSS: Non-Motor Symptom Scale; OH: Orthostatic Hypotension; PDQ-8: Parkinson’s Disease Questionaire-8; SCOPA-AUT: Scale for Outcomes in Parkinson’s Disease-Autonomic.
